# Trajectories of cardiac troponin in the decades before cardiovascular death: a longitudinal cohort study

**DOI:** 10.1186/s12916-023-02921-8

**Published:** 2023-06-19

**Authors:** Dorien M. Kimenai, Atul Anand, Marie de Bakker, Martin Shipley, Takeshi Fujisawa, Magnus N. Lyngbakken, Kristian Hveem, Torbjørn Omland, Carlos A. Valencia-Hernández, Joni V. Lindbohm, Mika Kivimaki, Archana Singh-Manoux, Fiona E. Strachan, Anoop S. V. Shah, Isabella Kardys, Eric Boersma, Eric J. Brunner, Nicholas L. Mills

**Affiliations:** 1 British Heart Foundation/University Centre for Cardiovascular Science, The University of Edinburgh, Edinburgh, EH16 4SA UK; 2 Department of Cardiology, Erasmus MC, University Medical Center Rotterdam, Rotterdam, the Netherlands; 3 Department of Epidemiology and Public Health, University College London, London, UK; 4 Department of Cardiology, Akershus University Hospital, Lørenskog, Norway; 5 K.G. Jebsen Center for Cardiac Biomarkers, Institute of Clinical Medicine, University of Oslo, Oslo, Norway; 6 Department of Public Health and General Practice, HUNT Research Centre, Norwegian University of Science and Technology, Levanger, Norway; 7 Levanger Hospital, Nord-Trøndelag Hospital Trust, Levanger, Norway; 8 Department of Public Health, University of Helsinki, Helsinki, Finland; 9 Epidemiology of Ageing and Neurodegenerative Diseases, Inserm U1153, Université de Paris, Paris, France; 10 Usher Institute, University of Edinburgh, Edinburgh, UK; 11 Department of Non-Communicable Disease, London School of Hygiene and Tropical Medicine, London, UK

**Keywords:** Cardiac troponin, Outcome, Risk factors, General population

## Abstract

**Background:**

High-sensitivity cardiac troponin testing is a promising tool for cardiovascular risk prediction, but whether serial testing can dynamically predict risk is uncertain. We evaluated the trajectory of cardiac troponin I in the years prior to a cardiovascular event in the general population, and determine whether serial measurements could track risk within individuals.

**Methods:**

In the Whitehall II cohort, high-sensitivity cardiac troponin I concentrations were measured on three occasions over a 15-year period. Time trajectories of troponin were constructed in those who died from cardiovascular disease compared to those who survived or died from other causes during follow up and these were externally validated in the HUNT Study. A joint model that adjusts for cardiovascular risk factors was used to estimate risk of cardiovascular death using serial troponin measurements.

**Results:**

In 7,293 individuals (mean 58 ± 7 years, 29.4% women) cardiovascular and non-cardiovascular death occurred in 281 (3.9%) and 914 (12.5%) individuals (median follow-up 21.4 years), respectively. Troponin concentrations increased in those dying from cardiovascular disease with a steeper trajectory compared to those surviving or dying from other causes in Whitehall and HUNT (*P*_interaction_ < 0.05 for both). The joint model demonstrated an independent association between temporal evolution of troponin and risk of cardiovascular death (HR per doubling, 1.45, 95% CI,1.33–1.75).

**Conclusions:**

Cardiac troponin I concentrations increased in those dying from cardiovascular disease compared to those surviving or dying from other causes over the preceding decades. Serial cardiac troponin testing in the general population has potential to track future cardiovascular risk.

**Supplementary Information:**

The online version contains supplementary material available at 10.1186/s12916-023-02921-8.

## Background

With approximately 17.6 million deaths each year, cardiovascular disease remains the leading cause of mortality worldwide [1]. Novel approaches to estimate risk are needed to help target effective primary prevention therapies to individuals at high risk of developing cardiovascular disease. High-sensitivity cardiac troponin assays have raised the possibility that troponin testing could be used to guide therapeutic approaches beyond diagnosis of myocardial infarction in those who have not yet developed symptomatic cardiovascular disease. Previous studies have consistently demonstrated that cardiac troponin is a strong independent predictor of future cardiovascular events in the general population [2–12].

How cardiac troponin testing should be used for cardiovascular risk estimation and whether serial testing would help track risk within individuals is uncertain. Lyngbakken et al*.* showed that the absolute and relative increases in cardiac troponin concentrations are independently associated with cardiovascular risk [13]. We demonstrated that cardiac troponin concentrations decline following statin therapy and the magnitude of the decline is an independent predictor of future cardiac events [3]. The dynamic behavior of cardiac troponin suggests it may be a sensitive and responsive risk marker that could be used in cardiovascular risk management, and the prevention of cardiovascular disease. However, the trajectory of cardiac troponin in the years prior to cardiovascular events is unknown, and insights here are needed to inform the role for serial measurements to track risk in practice.

Using the Whitehall II longitudinal cohort, our objective was to evaluate the trajectories of cardiac troponin I in middle aged individuals who died from cardiovascular disease compared to those who survived or died from non-cardiovascular causes during 21 years of follow up using retrospective trajectory analyses. Furthermore, we externally validate this analysis and determine prospectively whether the rate of change between cardiac troponin measurements is associated with risk of cardiovascular death, and if serial measuresments can be used to dynamically track cardiovascular risk within individuals.

## Methods

### Study population

The Whitehall II study is an ongoing longitudinal observational cohort of 10,308 civil servants based in London, who were first recruited in 1985 when aged between 35–55 years old [14]. Follow-up has continued over 12 phases, with the most recent assessment completed in 2016. Stored samples were available for cardiac troponin testing from participants assessed in 1997–1999, 2007–2009 and 2012–2013. We included participants who had at least one measure of cardiac troponin and outcome data, and we considered each participant’s first cardiac troponin measurement as baseline. That means that the first cardiac troponin measurement could have been taken in the time period 1997–1999, 2007–2009 or 2012–2013. For each participant, we collected clinical characteristics at baseline, and each participant’s first cardiac troponin measurement was defined as baseline. The study was approved by University College London Hospital Committee on Ethics of Human Research (reference 85/0938), and conducted according to declaration of Helsinki.

### Cardiac troponin measurements

Blood samples for each phase were handled according to a standardized protocol. Fasting venous blood samples were collected, centrifuged, and serum was stored in aliquots at − 80 °C until batch analysis was performed. Cardiac troponin I concentrations were measured using Siemens Atellica IM High Sensitivity Troponin I assay (Siemens Healthineers, Erlangen, Germany). This assay has a limit of blank of 0.5 ng/L, limit of detection of 1.6 ng/L and a limit of quantitation (LoQ) of 2.5 ng/L. The sex-specific 99^th^ percentiles are 34 ng/L and 53 ng/L in women and men, respectively.

### Clinical outcomes

Outcomes were collected throughout the study period until September 2019 using National Health Service (NHS) Central Registry [15]. Cardiovascular death, including coronary heart disease and stroke, was the primary outcome. Secondary outcomes were cardiac death, non-cardiovascular death, and all-cause death. Clinical outcomes were defined using the 9^th^ and 10^th^ revision of the International Classification of Diseases (ICD-9 and ICD-10): cardiovascular death (ICD 9: 340–459 or ICD-10: I00-I99), cardiac death (ICD 9: 410–414 or ICD-10: I20-I25) and non-cardiovascular death (all other ICD codes). Non-fatal myocardial infarction was defined using Hospital Episode Statistics (HES) database records up to March 2019 using ICD-9 and ICD-10 codes (ICD-9: 410 or ICD-10 codes: I21) where listed in primary or secondary position [16].

### Validation cohort

External validation was undertaken using the Trøndelag Health (HUNT) Study. The HUNT Study is a longitudinal observational population-based cohort study with over 120,000 individuals from Nord-Trøndelag county, as previously described [13, 17]. For this study, we included 9,711 individuals who had at least one cardiac troponin I measurement available (HUNT 2, 1995–1997) using the ARCHITECT i2000SR high-sensitivity cardiac troponin I assay (Abbott Diagnostics). This assay has a limit of blank of 0.7 ng/L, limit of detection 1.2 ng/L and LoQ of 3.2 ng/L [18]. The sex-specific 99^th^ percentiles are 16 ng/L in women and 34 ng/L in men [19]. Of these individuals, 5,337 (55.0%) had a second troponin measurement (HUNT 3, 2006–2008). Follow-up data was available until the end of December 2016. The HUNT Study was approved by Regional Committee for Medical Research Ethics (REC 2012/859, REC 2016/801) and Norwegian Data Inspectorate Board, and all participants provided informed written consent.

### Statistical analysis

Continuous variables are presented as mean ± standard deviation (SD) or median, 25^th^-75^th^ percentile, as appropriate. Categorical variables are presented as absolute number (%). For the primary analyses, cardiac troponin values below the limit of blank of 0.5 ng/L were assigned a value at the limit of blank.

We conducted analysis of trajectories to evaluate the temporal pattern of cardiac troponin prior to the primary outcome of cardiovascular death using linear mixed-effects modeling. Participants were classified according to whether they did or did not have a cardiovascular death during follow-up. Date of event, or end of follow-up in those where the event did not occur was considered as time point 0. In a backward fashion, cardiac troponin levels from each phase were used to estimate average cardiac troponin pattern of the groups over time using fixed-effect coefficients of the linear mixed-effects model. We applied log_2_ transformation to achieve normal distribution for troponin. Time was entered into fixed- and random effects part of the model. We used natural cubic splines to assess non-linear associations and final model was chosen based on lowest Akaike Information Criteria. The model was adjusted for age and sex and included an interaction term for cardiovascular death and time-to-event for estimation of average troponin trajectories stratified by event.

To evaluate whether baseline cardiac troponin level in combination with relative or absolute rate of change of cardiac troponin concentrations over time was prospectively associated with cardiovascular death, we classified those individuals where first two cardiac troponin measurements were obtained 10-years apart into the following four groups: Group 1 = baseline level ≤ LoQ and ≤ median change, Group 2 = baseline level ≤ LoQ and > median change, Group 3 = baseline level > LoQ and ≤ median change and Group 4 = baseline level > LoQ and > median change (LoQ = 2.5 ng/L, median relative change = 49.3% increase, median absolute change = 1.4 ng/L increase). For each group, we estimated cumulative incidence of cardiovascular death at 10 years after the second measurement and group comparisons were made using the log-rank test. Non-cardiovascular death was considered as competing risk.

Joint multistate modeling was used to evaluate the association between individual cardiac troponin trajectories and cardiovascular death. Joint modeling is based on the principle that it combines a linear mixed-effects model for repeated measurements of cardiac troponin with a time-to-event relative risk model for time-to-event data to evaluate the temporal pattern of a predictor in relation to hazard of an outcome (Additional file: Appendix) [20, 21]. Hence, a joint model relates the individual’s trajectory of cardiac troponin to his/her prognosis while accounting for different follow-up durations between individuals. In the context of repeated measurements, we not only studied the predictive value of cardiac troponin levels, but we also studied the predictive value of the slope of longitudinal trajectory (rate of change) and area under trajectory of cardiac troponin (Additional file: Appendix). The linear mixed-effects model was adjusted for sex and age. Crude and multivariable relative risk models were used with cardiovascular death as dependent variable. Non-cardiovascular death was included in the model as a competing risk to cardiovascular death. In the multivariable models, we adjusted for age and sex, and subsequently for known cardiovascular risk factors (sex, age, diabetes mellitus, total cholesterol levels, high-density lipoprotein levels, low-densitity lipoprotein levels, systolic blood pressure, and smoking status). The results are presented as hazard ratios (HRs) and 95% confidence intervals (95% CI) per doubling increase in cardiac troponin levels, slope (delta of the cardiac troponin’s levels/ 5 years) and area.

In secondary analyses, we assessed time trajectories of cardiac troponin in relation to cardiac death, non-cardiovascular death, death from any cause, fatal and non-fatal myocardial infarction. Furthermore, we investigated the association between individual cardiac troponin trajectories and hazard of cardiovascular death in a population without cardiovascular disease at baseline. We also explored the association of clinical characteristics at baseline (sex, age, ethnicity, diabetes status, systolic blood pressure, total cholesterol, high-density lipoprotein, low-density lipoprotein, smoking status, and body mass index) with repeated measures of cardiac troponin using univariable linear mixed-effects models. We illustrated how dynamic profiling of cardiovascular risk could be applied in practice while using individual cardiac troponin trajectories. As dynamic risk prediction using joint multistate models is still under development, we estimated an individual’s risk based on a non-competing joint model that was adjusted for known cardiovascular risk factors. Accuracy of the model was determined using a time-dependent area under curve methodology.

Single imputation was applied for clinical characteristics with missing values using other individual’s clinical and outcome data, and we used a single imputation technique where each incomplete variable was imputed by a separate model (fully conditional specification method). Statistical analysis was performed using R version 3.6.2. We used the packages *mice*, *nlme*, *survival* and *JMbayes2*.

## Results

### Clinical characteristics of study population

There were 7,293 individuals (29.4% women, 58 ± 7 years of age) with at least one cardiac troponin result available (Additional file: Fig. S1). The majority of individuals included in our study had their first troponin measurement in the time period 1997–1999 (6,028/7,293 [82.7%]). The remaining individuals had their first troponin measurement in 2007–2009 or 2012–2013 (1,117/7293 [15.3%], 148/7,293 [2.0%]), respectively. Of the total study population, 5,818 (79.8%) and 4,045 (55.5%) individuals had a second and third measurement, respectively (Additional file: Table S1). A total of 6,524 (89.5%), 5,694 (97.9%) and 3,996 (98.8%) individiuals had detectable cardiac troponin concentrations at their first, second and third measurement, respectively. The median cardiac troponin concentration was lowest at baseline (3.3 ng/L [25^th^-75^th^ percentile 2.2–5.3 ng/L]) and increased over time (second measurement, 4.6 ng/L [25^th^-75^th^ percentile 3.2–7.3 ng/L]: third measurement, 5.2 ng/L [25^th^-75^th^ percentile 3.6–8.1 ng/L], Table 1). This was observed in all age groups and when stratified by sex (Additional file: Fig. S2). Sex and age, but not ethnicity, was associated with longitudinal cardiac troponin I (Additional file: Table S2). We also found signifant associations with conventional cardiovascular risk factors and repeated measures of cardiac troponin. Cardiovascular death occurred in 281 (3.9%) individuals during a median follow-up of 21.4 [25^th^-75^th^ percentile, 15.8–21.8] years (Table 1). Cardiac death, non-cardiovascular death and all-cause death occurred in 143 (2.0%), 914 (12.5%), and 1,195 (16.4%) individuals, respectively (Additional file: Table S3).

**Table 1:** Baseline characteristics

Clinical characteristic	Study population (*n* = 7,293)	No cardiovascular death (*n* = 7,012)	Cardiovascular death (*n* = 281)
Sex (female)	2,142 (29.4%)	2,066 (29.5%)	76 (27.0%)
Age (years)	58 (7)	57 (7)	61 (6)
Ethnic origin (other than white)	625 (8.6%)	585 (8.4%)	40 (14.2%)
Diabetes mellitus (yes)	333 (4.6%)	302 (4.3%)	31 (11.0%)
Systolic blood pressure (mmHg)	124 (17)	124 (17)	129 (18)
Total cholesterol (mmol/L)	5.8 (1.1)	5.8 (1.1)	5.9 (1.2)
High-density lipoprotein (mmol/L)	1.5 (0.4)	1.5 (0.4)	1.4 (0.4)
Low-density lipoprotein (mmol/L)	3.7 (1.0)	3.7 (1.0)	3.8 (1.0)
Smoker			
Never	3,451 (48.5%)	3,328 (48.6%)	123 (44.9%)
Ex-smoker	3,000 (42.2%)	2,885 (42.2%)	115 (42.0%)
Current	665 (9.3%)	629 (9.2%)	36 (13.1%)
Body mass index (kg/m^2^)	26.4 (4.2)	26.3 (4.1)	27.5 (4.9)
Lipid-modifying medication (yes)	616 (8.5%)	585 (8.4%)	31 (11.2%)
Antihypertensive medication (yes)	1,267 (17.5%)	1,165 (16.8%)	102 (36.8%)
ACE inhibitors (yes)	561 (7.8%)	525 (7.5%)	36 (13.0%)
Antiplatelets (yes)	518 (7.2%)	473 (6.8%)	45 (16.2%)
Betablockers (yes)	448 (6.2%)	409 (5.9%)	39 (14.1%)
Troponin I level at baseline (ng/L)	3.3 [2.2 to 5.3]	3.3 [2.2 to 5.1]	4.8 [3.0 to 9.3]
Troponin I level at second measurement (ng/L)^a^	4.6 [3.2 to 7.3]	4.6 [3.2 to 7.2]	7.4 [5.1 to 15.2]
Troponin I level at third measurement (ng/L)^b^	5.2 [3.6 to 8.1]	5.1 [3.6 to 8.1]	8.4 [5.9 to 18.0]

Continuous variables are presented as mean (SD) or median (25^th^ to 75^th^ percentile), as appropriate. Categorical variables are presented as number (%)

*Abbreviations*: *ACE* Angiotensin-converting enzyme. Proportion missing values: no missing values except for ethnicity (0.2%), systolic blood pressure (0.2%), high-density lipoprotein (9.3%), low-density lipoprotein (10.4%), smoking (2.4%), lipid-modifying medication (0.9%), antihypertensive medication (0.9%), ACE inhibitors (0.9%), antiplatelets (0.9%), betablockers (0.9%) and body mass index (11.4%)

^a^Individuals, *n* = 5,818, median (25^th^ to 75^th^ percentile) time between baseline and second troponin measurement: 10.4 (10.0–10.8) years

^b^Individuals, *n* = 4,045, median (25^th^ to 75^th^ percentile) time between baseline and third troponin measurement: 14.6 (14.4–14.6) years

In those who experienced the primary outcome of cardiovascular death, we observed a higher baseline risk profile, as they were older (61 ± 6 *versus* 57 ± 7 years, *P* < 0.001), more likely to have diabetes (11.0% *versus* 4.3%, *P* < 0.001) and have a higher systolic blood pressure (129 ± 18 mmHg *versus* 124 ± 17 mmHg, *P* < 0.001). The group with cardiovascular death were also prescribed more cardiac medications including ACE inhibitors (13.0% *versus* 7.5%, *P* = 0.001), antihypertensives (36.8% *versus* 16.8%, *P* < 0.001) and beta-blockers (14.1% *versus* 5.9%, *P* < 0.001).

### Cardiac troponin trajectories in relation to cardiovascular death

**Fig. 1:**
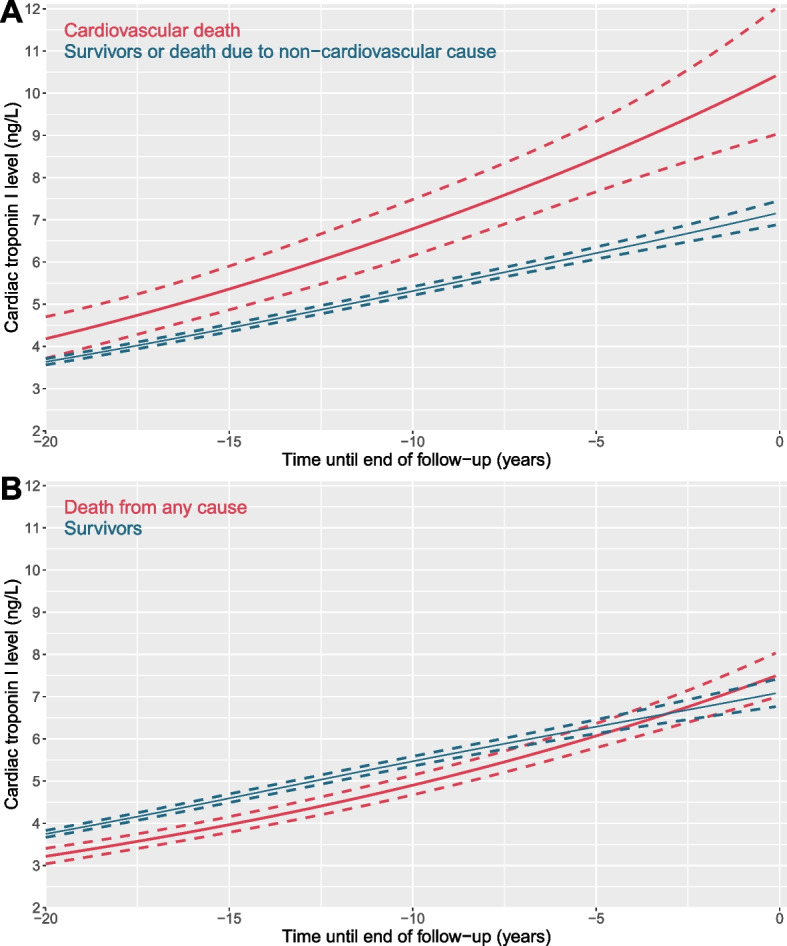
Average trajectories of troponin I with 95% CIs before cardiovascular death and death from any cause (*n* = 7,293). **A**, red line refers to troponin trajectory of those individuals who died due to cardiovascular causes, and the blue line refers to troponin trajectory of those individuals who survived or died due to non-cardiovascular causes. **B**, red line refers to troponin trajectory of those individuals who died from any cause, and the blue line refers to troponin trajectory of those individuals who survived

We evaluated cardiac troponin trajectories in relation to cardiovascular death in all 7,293 individuals. At baseline, cardiac troponin concentrations were higher in the individuals who died from cardiovascular disease as compared to those who did not (5 [25^th^-75^th^ percentile, 3–9] ng/L *versus* 3 [25^th^-75^th^ percentile, 2–5] ng/L, *P* < 0.001, Table 1). Cardiac troponin concentrations were higher in those dying from cardiovascular disease and they had a steeper trajectory as compared to those surviving or dying from other causes over the preceding decades (*P*_*interaction*_ time*cvd death = 0.037, Fig. 1A). In contrast, we did not observe a significant difference in temporal pattern of cardiac troponin between those who experienced death from any cause (*P*_*interaction*_ time*all-cause death = 0.055, Fig. 1B) or non-cardiovascular death (*P*_*interaction*_ time*non-cvd death = 0.364, Additional file: Fig. S3-S4) as compared with individuals who did not. Higher cardiac troponin concentrations were observed over time in individuals who died due to cardiac disease compared to those who did not, but the trajectory of cardiac troponin was similar (*P*_*interaction*_ time*chd death = 0.827, Additional file: Figure S5). Cardiac troponin concentrations were higher in individuals who experienced fatal myocardial infarction with no steeper trajectory observed as compared to individuals who did not experience the event (*P*_*interaction*_ time*fatal MI = 0.510), while we did not observe higher cardiac troponin concentrations in those who experienced a non-fatal myocardial infarction event (*P*_*interaction*_ time*non-fatal MI = 0.127, Additional file: Figure S6).

### Rate of change and individual cardiac troponin trajectories in relation to cardiovascular death

**Fig. 2:**
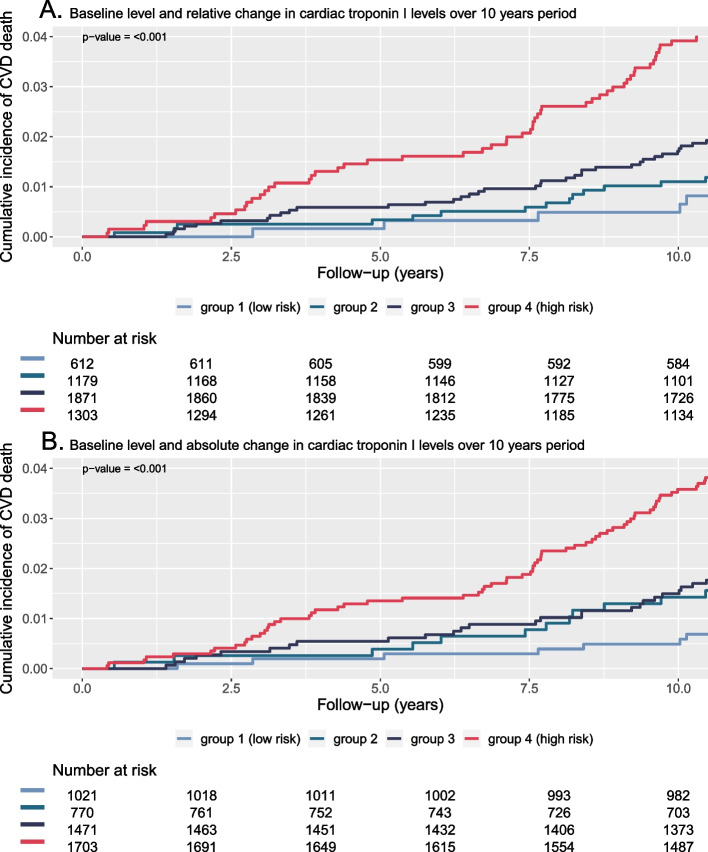
Baseline level and relative (**A**) or absolute change (**B**) in troponin levels and cardiovascular death (*n* = 4,695). Group 1 = baseline level ≤ LoQ and ≤ median change, Group 2 = baseline level ≤ LoQ and > median change, Group 3 = baseline level > LoQ and ≤ median change, Group 4 = baseline level > LoQ and > median change

We evaluated the absolute cardiac troponin level at baseline combined with the relative- and absolute change in cardiac troponin levels over a 10-year period in relation to the cumulative incidence of cardiovascular death. In 4,965 individuals, we had two troponin measurements available with a 10-year time frame between the first two measurements. We observed that those with lowest cardiac troponin level at baseline and with the lowest relative- or absolute cardiac troponin increase were at lowest risk, and those with highest cardiac troponin level at baseline and with highest relative- or absolute cardiac troponin increase were at highest risk (Fig. 2*,* *P* < 001 for both relative- and absolute rate of change groups). Cumulative incidence of cardiovascular death at 10 years between relative- and absolute rate of change groups was similar (Group 1 to 4 for baseline cardiac troponin level combined with relative change over time: 0.5%, 1.1%, 1.7%, 3.9% (Fig. 2A); Group 1 to 4 for cardiac troponin level combined with absolute change over time: 0.5%, 1.4%, 1.5%, 3.6% (Fig. 2B)).

Accordingly, we evaluated in all individuals (n = 7,293) the relationship of individual cardiac troponin trajectories and cardiovascular death using the multi-state joint model approach. Using individual cardiac troponin trajectories, we showed that doubling in cardiac troponin levels at any point in time during follow-up was associated with a higher risk of cardiovascular death (unadjusted HR 1.53, 95% CI 1.32–1.75, *P* < 0.001, Table 2). The association persisted when we included known cardiovascular risk factors in the model (adjusted HR 1.45, 95% CI 1.32–1.58, *P* < 0.001, Table 2, Additional file: Table S4). The slope in cardiac troponin was not associated with cardiovascular death (unadjusted HR 0.89, 95% CI 0.35–2.29, *P* = 0.809). In other words, independent of the cardiac troponin level, a doubling of the slope over 5-years at any point during follow-up does not differentiate between an individual with and without a cardiovascular death. In contrast, the area under trajectory of cardiac troponin at any point in time was independently associated with cardiovascular death with an adjusted HR of 1.41 (95% CI 1.29–1.53, *P* < 0.001) per doubling. The findings were consistent, although slightly attenuated, in a population without cardiac disease at baseline showing a significant association between the cardiac troponin trajectory and cardiovascular death (Additional file: Fig. S7, Table S5-S6).

**Table 2:** Association between the temporal evolution of cardiac troponin I and cardiovascular death

	**HR (95% CI)**	***P*-value**
**Crude Model**^**a**^		
Temporal evolution of cardiac troponin I^b^		
Level	1.53 (1.32 to 1.75)	< 0.001
Slope	0.89 (0.35 to 2.29)	0.809
Area	1.53 (1.32 to 1.74)	< 0.001
**Adjusted model (age and sex)**^**a**^		
Temporal evolution of cardiac troponin I^b^		
Level	1.47 (1.34 to 1.60)	< 0.001
Slope	0.95 (0.40 to 2.36)	0.893
Area	1.46 (1.34 to 1.60)	< 0.001
Age, years	1.10 (1.08 to 1.13)	< 0.001
Sex, female	1.06 (0.80 to 1.40)	0.663
**Adjusted model (known CVD risk factors**^**c**^**)**^**a**^		
Temporal evolution of cardiac troponin I^b^		
Level	1.45 (1.32 to 1.58)	< 0.001
Slope	0.97 (0.41 to 2.30)	0.947
Area	1.41 (1.29 to 1.53)	< 0.001
Age, years	1.10 (1.07 to 1.13)	< 0.001
Sex, female	1.04 (0.76 to 1.40)	0.786
Diabetes mellitus, yes	2.41 (1.57 to 3.60)	< 0.001
Total cholesterol, mmol/L	1.15 (0.86 to 1.51)	0.360
High-density lipoprotein, mmol/L	0.73 (0.51 to 1.06)	0.096
Low-density lipoprotein, mmol/L	0.93 (0.69 to 1.29)	0.666
Systolic blood pressure, 10 mmHg	1.09 (1.01 to 1.16)	0.025
Smoking status, current	1.95 (1.34 to 2.78)	0.001

^a^Individuals, *n* = 7,293

^b^Hazard ratios (HRs) and 95% confidence intervals (CIs) are given per doubling in cardiac troponin in level, slope (delta of the cardiac troponin’s levels/ 5 year) and area under the trajectory of cardiac troponin

^**c**^The model adjusted for known cardiovascular risk factors included age, sex, diabetes mellitus, total cholesterol, high-density lipoprotein, low-density lipoprotein, systolic blood pressure, smoking status and serial cardiac troponin measurements

### Dynamic profiling of cardiovascular risk using individual cardiac troponin trajectories

**Fig. 3:**
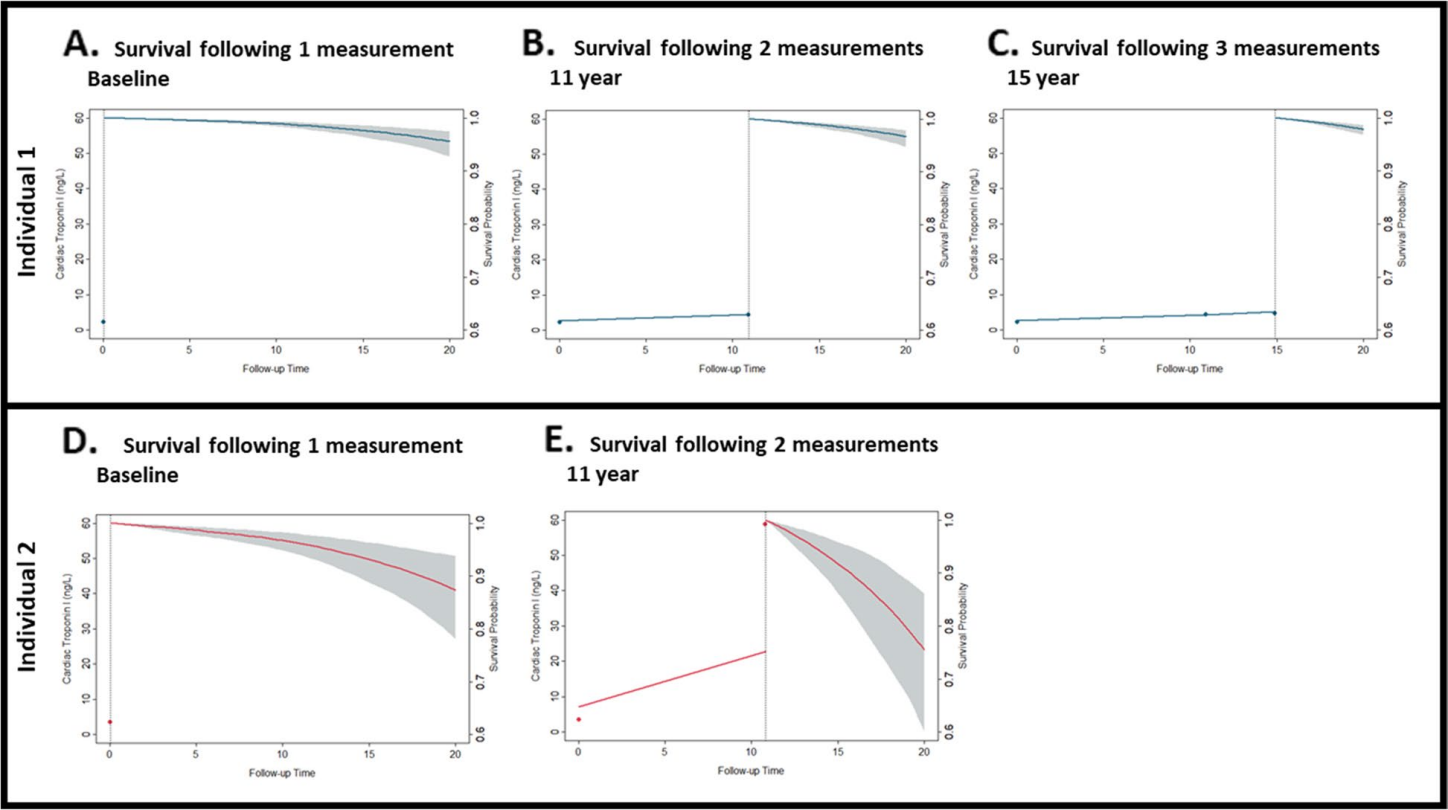
Dynamic cardiovascular risk profiling using individual troponin trajectories. Dynamic profiling of an individual’s risk using troponin trajectory for an individual with (**A-C**, blue line) and without stable cardiac troponin levels (**D**,** E**, red line) over time. The individual-specific cardiac troponin trajectory (per ng/L) is shown on the left Y-axis and survival probability with 95% confidence intervals (%) is shown on the right Y-axis. Up to three measurements, the survival probability curve is dynamically updated

Here we illustrate two examples of dynamic profiling of cardiovascular risk using serial cardiac troponin measurements. The accuracy of models to dynamically profile cardiovascular risk using individual cardiac troponin trajectories is reported in Additional file: Table S7. We estimated the probability of survival for a woman who was 64 years old, who had no diabetes and was a non-smoker, and who had stable cardiac troponin measurements over time (Fig. 3A-C). With her initial cardiac troponin measurement of 2 ng/L, her conditional survival probability to reach the 20-years follow-up period was 95.5%. Her conditional survival probability remained similar when the risk model was updated with three measurements (second measurement, 5 ng/L at 10.9 years, third measurement, 5 ng/L at 14.9 years; survival probability of 97.8%). Additionally, we estimated the survival probability of a man with an initial low cardiac troponin level of 3 ng/L that increased to 59 ng/L at 10.8 years (Fig. 3D, E). The man was 64 years old, had no diabetes mellitus and was a current smoker. When only using information of first troponin measurement, his estimated survival probability for reaching the 20-years follow-up period was 87.4%, and with updated information of the second measurement, the survival probability declined to 75.5%.

### External validation

The external validation cohort has been described previously [13, 17]. Of the 9,711 individuals (54.4% women, 50 ± 17 years of age) included in this analysis, 5,337 (55.0%) had a second troponin measurement after a 10-year period. During a median follow-up of 19.9 (25^th^-75^th^ percentile, 19.8–20.2) years, 1,102 (11.3%) cardiovascular deaths were registered. Consistent with trajectory analysis in Whitehall II cohort, we observed that the cardiac troponin trajectories were different in those individuals who died due to cardiovascular disease as compared to those who survived or died due to non-cardiovascular causes (*P*_*interaction*_ time*cvd death = 0.049, Additional file: Fig. S8). As only two cardiac troponin measurements were available, joint modeling analysis to evaluate the individual trajectories of cardiac troponin in relation to cardiovascular death was not possible. Instead, a time-dependent Cox regression analysis was conducted to evaluate the association with serial cardiac troponin measurements and cardiovascular death. Serial measurements of cardiac troponin was independently associated with cardiovascular death (adjusted HR 1.33, 95% CI 1.25–1.40, *P* < 0.001, Additional file: Table S8).

## Discussion

Using serial measurements, we evaluated the relationship between the trajectory of cardiac troponin I and cardiovascular death over 20 years. Our study has two main findings. First, in a retrospective analysis individuals who died from cardiovascular disease had different cardiac troponin trajectories with higher and increasingly divergent concentrations over two decades prior to death, when compared to those who survived or died from non-cardiovascular causes. In contrast, there was no difference in the cardiac troponin trajectory or concentrations in those with non-cardiovascular or death from any cause. Second, using joint modeling techniques we demonstrate that cardiac troponin can be used to track risk dynamically over time within an individual, raising the possibility that serial high-sensitivity measurements could be used to monitor and refine estimates of cardiovascular risk in the general population.

Our study has three main strengths and areas of novelty. First, our study is the first to assess cardiovascular risk using the Siemens Atellica high-sensitivity cardiac troponin I assay outside the setting of acute coronary syndrome [22]. Second, the trajectory of cardiac troponin prior to cardiovascular events was unknown. We provide the first description by harnessing three serial measurements and 20 years of follow up to demonstrate that the cardiac troponin trajectories of individuals who die from cardiovascular disease differ from those who survive or die to due non-cardiovascular conditions. Furthermore, we validate this observation in an external cohort using a different cardiac troponin assay confirming that our findings are generalisable. Third, the use of repeated cardiac troponin measurements for cardiovascular risk estimation has been limited by the lack of availability of suitable modeling techniques that can account for missing data and competing risks from non-cardiovascular death. To date only the BiomarCaRE study has applied joint modelling to evaluate the association between repeated cardiac troponin measurements and cardiovascular outcomes [23]. Since this analysis joint model techniques have undergone further development and it has become possible to account for competing risk. In our study, we have used multistate joint modeling to address competing risk and relate cardiac troponin trajectories to a range of important outcomes within an individual. Our approach goes beyond traditional static prediction models such as the Framingham risk score and SCORE2 [24, 25], and enables dynamic tracking of cardiovascular risk over time. Together our observations and novel methodological approach provide insights that will inform the development of the next generation of cardiovascular risk estimation systems.

The association between cardiac troponin measured at a single timepoint and future cardiovascular risk is well established [26, 27]. Welsh et al*.* demonstrated a 56% increase in the risk of cardiovascular death over a median 7.8 years of follow-up for each standard deviation increase in cardiac troponin I, after adjustment for age, sex and known cardiovascular risk factors [2]. Similar observations were noted within the BiomarCaRE project [7]. These findings suggest a potentially important role for cardiac troponin measurement in cardiovascular risk assessment. However, if this approach were implemented it is likely that serial measurements will be performed in practice but few studies have evaluated changes within an individual [28].

The HUNT Study included 4,805 participants with two measures of high-sensitivity cardiac troponin I separated by 10 years [13]. Participants were categorized into three groups according to change in cardiac troponin with both absolute and relative change related to increased cardiovascular death, myocardial infarction or heart failure [13]. This is broadly consistent with our findings from up to three samples over a longer follow-up period. Similar relationships with cardiovascular risk were noted in the WOSCOPS study, but this was an exclusively male population with just one year between the two cardiac troponin measures [3]. However, the methods of classification applied in both studies have limited value for individual risk prediction, where identifying someone as ‘low’ or ‘high’ risk is too simplistic. Precision medicine advocates for individualized estimates of risk that may be updated as further information, including additional cardiac troponin measurements, become available. With further development our proposed dynamic risk estimation system and serial measurements to update estimates of cardiovascular risk could provide a more individualised approach. A dynamic measure of risk could encourage patients to remain on therapy, or guide clinicians to escalate therapy where risk remains elevated. In previous work, we demonstrated that cardiac troponin concentrations decline following the initiation of statin therapy, and the magnitude of this decline was an independent predictor of future cardiac events, even after adjustment for baseline or change in cholesterol concentration [3]. Incorporating serial measures of cardiac troponin into a dynamic risk estimation system would represent a substantial change in approach to the prevention of cardiovascular disease with the potential to improve outcomes and provide additional public health benefits.

We propose joint modeling as a novel approach that uses individual troponin trajectories to estimate risk. Although we acknowledge that our study only shows a conceptual design of this technique at present. A dynamic risk estimation system would represent a substantial change in approach to the prevention of cardiovascular disease, but such an approach would have major potential to improve outcomes and provide additional public health benefits. We recommend that a comprehensive evaluation on risk factor selection should take place on the trajectories of both traditional and non-traditional risk factors for dynamic prediction of cardiovascular risk. Further development including serial measures of lifestyle factors (e.g., diet and physical activity), traditional cardiovascular risk factors (e.g., blood pressure and cholesterol), non-traditional risk factors (e.g., social deprivation) and other biomarkers (e.g.. N-terminal pro-B-type natriuretic peptide, C-reactive protein and creatinine), could create a more specific, interactive and dynamic clinical tool to improve cardiovascular risk prediction. It would be important to identify which of these markers benefit from serial measurement and to identify the optimum frequency. As the availability of cardiac imaging increases with automated methods for the quantification of coronary plaque it may become feasible to further stratify risk using imaging biomarkers. As the release and clearance of cardiac troponin is a dynamic process [29, 30], further research is required to identify the optimum time for blood sampling when serial measures of cardiac troponin or other biomarkers are obtained for cardiovascular risk prediction. Furthermore, we showed previously in WOSCOPS that cardiac troponin concentrations decline following statin therapy [3], and future research on the impact of preventative therapies on biomarker trajectories is necessary to ensure optimal precision for cardiovascular risk estimates. Further research is also needed to determine whether multistate joint models can provide dynamic risk prediction for composite endpoints that combine fatal and non-fatal cardiovascular events and how this should be applied in practice. The additional value of a dynamic risk estimation system over a static risk estimation system cannot be captured by a single performance metric and needs a broader perspective encompassing quantitative (c-statistic, calibration plots, brier score) and qualitative (behavioural change and adherence) measures.

The MORGAM/BiomarCaRE study also evaluated serial cardiac troponin testing for primary prevention in the general population [23]. Although they did not perform a trajectory analysis or undertake dynamic profiling on an individual level, their observations from joint modelling were broadly consistent with ours suggesting greater importance for the cardiac troponin level than rate of change over time. However, they observed that discriminative ability improved with updated information provided by repeated measurement. In both studies three cardiac troponin measurements were available over a 15-years time period. It is possible that more frequent sampling than available in our study would improve precision of our calculation of slope within our model and further improve our estimation of individual risk [31]. In clinical practice more frequent measurements would be needed to guide an individual’s risk and management than once every 10 years. Electronic patient record systems also allow the assimilation of multiple measurements, including important non-laboratory parameters, within risk estimation tools to enable dynamic monitoring of cardiovascular risk using joint modelling in practice. We cannot address in our analysis how often serial measurements of a biomarker or risk factor should be obtained for optimal performance of a dynamic risk estimation system to predict cardiovascular disease. Future work is required to determine the impact of increasing the frequency of measurement on performance.

It is assumed that detectable cardiac troponin concentrations in apparently healthy individuals are due to cardiomyocyte apoptosis or necrosis [32]. However, cardiac troponin release may also occur in the setting of reversible myocardial ischemia [33]. Furthermore, associations have been observed between cardiac troponin concentrations and a range of cardiac- and non-cardiac conditions other than coronary heart disease or myocardial infarction [34, 35]. In our study, we demonstrate that cardiac troponin concentrations increase as individuals age, even in those without cardiovascular events. This is important for the proposed clinical translation of high-sensitivity cardiac troponin where thresholds have been defined that identify individuals as low- or high-risk of future cardiovascular events [36]. Such thresholds would be challenging to apply in older populations, where specificity would be compromised by the trajectories observed in our study. It is also important to recognise that cardiac troponin concentrations may decrease following effective therapy or lifestyle changes [3, 37]. A threshold approach will always be inferior compared to the use of dynamic models that incorporate continuous measures and serial testing. We observed a significant association with sex and repeated measures of cardiac troponin indicating that a sex-specific approach may be required. Also associations with other important cardiovascular risk factors are found. To understand the clinical implications of our findings, additional research is needed into the underlying mechanisms of cardiac troponin release with age prior to the onset of cardiovascular disease. These studies would benefit from serial assessments with cardiac imaging and additional biomarkers, such as high-sensitivity C-reactive protein.

Our study has several limitations. First, cardiac troponin measurements were available over a 15-year period and the time between last cardiac troponin measurement and end of follow-up was on average 5 years. Therefore, trajectory analysis involved extrapolation of the linear-mixed effects models in indidivuals who survived. Second, up to three cardiac troponin measurements were available for an individual and it is likely that more repeated measures would have led to a higher precision of estimated cardiac troponin trajectories. Third, external validation of our joint model using the Siemens high-sensitivity cardiac troponin I assay is required prior to implementation into practice. However, no other longitudinal population-based cohorts with this specific assay were available which precluded an external validation for this assay at this point. We were able to externally validate our findings in HUNT using another high-sensitivity cardiac troponin I assay, and we demonstrated that for both assays the trajectories of cardiac troponin prior to cardiovascular death differed to those individuals who died due to non-cardiovascular disease or survivors. Fourth, it should be acknowledged that we have evaluated two high-sensitivity assays and our findings cannot be extended to other high-sensitivity cardiac troponin assays. Future work on the development of dynamic risk models should assess whether cardiac troponin measures could be standardised within the model so that it could be applied across different health care systems using different assays. Fifth, assay imprecision at low concentrations may impact on model performance, however only 10.5% of persons had initial cardiac troponin concentrations below the limit of detection. Sixth, our participants are largely Caucasian in origin and men are overrepresented in the study. Validation in other ethnic groups with more female participants is needed to broaden generalizability. Seventh, evaluation of baseline level and rate over change over 10-year period was restricted to individuals who had two measures available which has introduced immortal bias for this analysis. Finally, it is possible that participants with positive health seeking behaviours were more likely to remain within the longitudinal study and have repeated measurements, which may have introduced a bias.

## Conclusions

In conclusion, cardiac troponin I concentrations increase in those dying from cardiovascular disease compared to those surviving or dying from other causes over the preceding decades. Serial cardiac troponin testing with joint modeling techniques have potential to dynamically track cardiovascular risk within individuals in the general population.

## Supplementary Information

**Additional file 1:** **Supplementary Appendix.** Principle of joint modelling. **Table S1.** Clinical characteristics of study population at first, second and third troponin measurement. **Table S2.** Association between clinical characteristics and longitudinal cardiac troponin I. **Table S3.** Baseline characteristics of those who experienced an event. **Table S4.** Association between the cardiac troponin I level at baseline and cardiovascular death. **Table S5.** Baseline characteristics of individuals without cardiac disease at baseline. **Table S6.** Association between the temporal evolution of cardiac troponin I and cardiovascular death in individuals without cardiac disease at baseline. **Table S7.** The longitudinal cardiac troponin’s accuracy. **Table S8.** Association between serial cardiac troponin measurements and cardiovascular death in HUNT. **Fig S1.** Study flow diagram. **Fig S2.** Cardiac troponin I levels at baseline, 10 years and 15 years, stratified by sex and age groups. **Fig S3.** Trajectories of cardiac troponin I with 95% confidence intervals before non-cardiovascular death occurred or at end of follow-up. **Fig S4.** Trajectories of cardiac troponin I with 95% confidence intervals before cardiovascular death or non-cardiovascular death occurred or at end of follow-up. **Fig S5.** Trajectories of cardiac troponin I with 95% confidence intervals before cardiac death occurred or at end of follow-up. **Fig S6.** Trajectories of cardiac troponin I with 95% confidence intervals before non-fatal myocardial infarction, fatal myocardial infarction and no myocardial infarction event. **Fig S7.** Trajectories of cardiac troponin I with 95% confidence intervals in individuals without baseline cardiac disease before cardiovascular death occurred or at end of follow-up. **Fig S8.** Trajectories of cardiac troponin I with 95% confidence intervals before cardiovascular death and death from any causein HUNT.

## Data Availability

Because of the sensitive nature of the data collected for this study, requests to access the dataset from qualified researchers trained in human subject confidentiality protocols should be sent to Whitehall management team at  email: whitehall2@ucl.ac.uk.

## Abbrevations

ACE: Angiotensin-converting enzyme inhibitors
CI: Confidence interval
HES: Hospital episode statistics
HR: Hazard ratio
HUNT: The Trøndelag Health Study
ICD: International classification of diseases
LoQ: Limit of quantitation
NHS: National health service
SD: Standard deviation
