# Supplementary material for: Trajectories of cardiac troponin in the decades before cardiovascular death: a longitudinal cohort study
Source: BMC Med. 2023 Jun 19;21:216. doi: 10.1186/s12916-023-02921-8 (PMC10280894; doi:10.1186/s12916-023-02921-8)
Supplement: Supplementary file 1 — Additional file 1: Supplementary Appendix. Principle of joint modelling. Table S1. Clinical characteristics of study population at first, second and third troponin measurement. Table S2. Association between clinical characteristics and longitudinal cardiac troponin I. Table S3. Baseline characteristics of those who experienced an event. Table S4. Association between the cardiac troponin I level at baseline and cardiovascular death. Table S5. Baseline characteristics of individuals without cardiac disease at baseline. Table S6. Association between the temporal evolution of cardiac troponin I and cardiovascular death in individuals without cardiac disease at baseline. Table S7. The longitudinal cardiac troponin’s accuracy. Table S8. Association between serial cardiac troponin measurements and cardiovascular death in HUNT. Fig S1. Study flow diagram. Fig S2. Cardiac troponin I levels at baseline, 10 years and 15 years, stratified by sex and age groups. Fig S3. Trajectories of cardiac troponin I with 95% confidence intervals before non-cardiovascular death occurred or at end of follow-up. Fig S4. Trajectories of cardiac troponin I with 95% confidence intervals before cardiovascular death or non-cardiovascular death occurred or at end of follow-up. Fig S5. Trajectories of cardiac troponin I with 95% confidence intervals before cardiac death occurred or at end of follow-up. Fig S6. Trajectories of cardiac troponin I with 95% confidence intervals before non-fatal myocardial infarction, fatal myocardial infarction and no myocardial infarction event. Fig S7. Trajectories of cardiac troponin I with 95% confidence intervals in individuals without baseline cardiac disease before cardiovascular death occurred or at end of follow-up. Fig S8. Trajectories of cardiac troponin I with 95% confidence intervals before cardiovascular death and death from any causein HUNT. [file 12916_2023_2921_MOESM1_ESM.pdf]

SUPPLEMENTARY APPENDIX

Trajectories of cardiac troponin in the decades before cardiovascular death: a longitudinal cohort study

Dorien M Kimenai[1*], Atul Anand[1*], Marie de Bakker[2], Martin Shipley[3], Takeshi Fujisawa[1], Magnus N Lyngbakken[4,5], Kristian Hveem[6,7], Torbjørn Omland[4,5], Carlos A Valencia-Hernández[3], Joni V Lindbohm[3,8], Mika Kivimaki[3], Archana Singh-Manoux[3,9], Fiona E Strachan[10], Anoop SV Shah[11], Isabella Kardys[2], Eric Boersma[2], Eric J Brunner[3], Nicholas L Mills[1,10]

Running title: Kimenai et al. Troponin trajectories and CVD death

[1] BHF Centre for Cardiovascular Science, University of Edinburgh, Edinburgh, United Kingdom
[2] Department of Cardiology, Erasmus MC, University Medical Center Rotterdam, Rotterdam, the Netherlands
[3] Department of Epidemiology and Public Health, University College London, London, United Kingdom
[4] Department of Cardiology, Akershus University Hospital, Lørenskog, Norway
[5] K.G. Jebsen Center for Cardiac Biomarkers, Institute of Clinical Medicine, University of Oslo, Oslo, Norway
[6] HUNT Research Centre, Department of Public Health and General Practice, Norwegian University of Science and Technology, Levanger, Norway
[7] Levanger Hospital, Nord-Trøndelag Hospital Trust, Levanger, Norway
[8] Department of Public Health, University of Helsinki, Helsinki, Finland
[9] Epidemiology of Ageing and Neurodegenerative Diseases, Inserm U1153, Université de Paris, Paris, France
[10] Usher Institute, University of Edinburgh, Edinburgh, United Kingdom
[11] Department of non-communicable disease, London School of Hygiene and Tropical Medicine, London, United Kingdom

[*]These authors contributed equally

Supplementary Appendix

Joint modelling

The principle of joint modelling

To evaluate the association between a longitudinal marker (i.e., a measurement repeatedly monitored over time [cardiac troponin]), and the occurrence of an event over time (i.e. cardiovascular death), a joint model can be used. The joint model uses a separate regression model to describe the evolution of the marker over time and uses these estimated evolutions in a time-to-event relative risk model for the event of interest. A linear mixed-effects model is used to analyze the longitudinal marker over time, which results in an estimated level of the marker at each point in time, instead of assuming a constant level of the longitudinal marker between observed measurements. In the joint model, this estimated evolution is related to the event status (i.e., the estimated cardiac troponin I at the time of the event is used for the relative risk analysis, Figure 1A). For the joint modelling approach at least one measurement of a predictor variable is required per outcome and per individual and it does not matter which measurement is used (i.e., at baseline or at a later time point). Provided the above condition holds, the joint model works with all available data per outcome. Implicitly the missing data are imputed by the model under the missing not at random (MNAR) assumption, with specific missing data mechanism implied by joint models

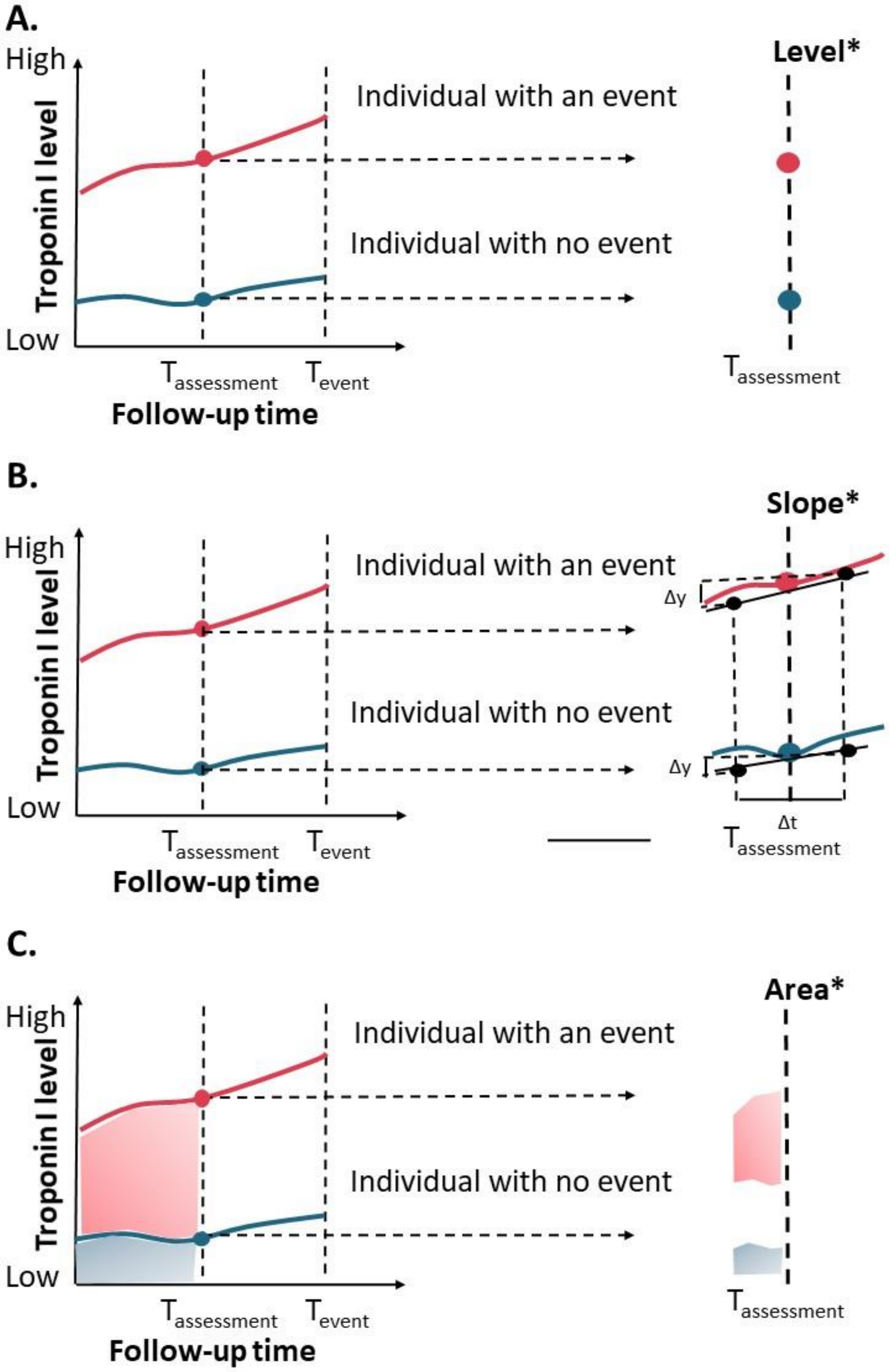

Figure 1. Joint modeling using repeated cardiac troponin I measurements. Illustrative presentation of the principle of joint modeling, and the type of parametrizations that can be evaluated based on the troponin trajectories. The figure shows trajectories for a fictive individual who does (red line) and who doesn't experience (green line) the event. Panel A: level of troponin at any point in time, Panel B: slope of troponin trajectory at any point in time, Panel C: Area under the trajectory of troponin up to the same point in time. *Hazard Ratio's that are derived from the joint model present the hazard of cardiovascular death of level, slope or area at any point in time.

Apart from relating the level of the marker to the risk of the event, the joint modeling framework allows for extensions assessing additional associations. Perhaps it is not (only) the level of the marker that is related to the event but the fact that the marker is increasing rapidly at that moment. This would be of particular interest in situations where, for example, at a specific point in time two individuals show similar marker levels, but different rate of change of the marker. The rate of change of a longitudinal marker (i.e., the slope of the marker at that moment in time), can be added to the joint model to analyze its relationship with the event of interest. The slope, as evaluated by the joint model, indicates whether and by how much a marker is increasing or decreasing at any moment (Figure 1B), which differs from the absolute (or relative) rate of change that corresponds to the constant rate of change between two time points (Figure 2). In our study, we observed a non-significant relationship with the slope of cardiac troponin the hazard of the event. The level of cardiac troponin remains significant, so we can conclude that the velocity of the concentration has no significant additional information on top of its absolute level at each point in time. Incorporating troponin rate of change together with absolute concentrations may give additional information on the risk of cardiovascular death, although absolute concentrations appear the driving factor for predicting cardiovascular death. Apart from the level and slope of the longitudinal marker, multiple other features can easily be included in the joint model, for instance: area under the curve (if the cumulative burden of a marker has an effect on the event, Figure 1C).

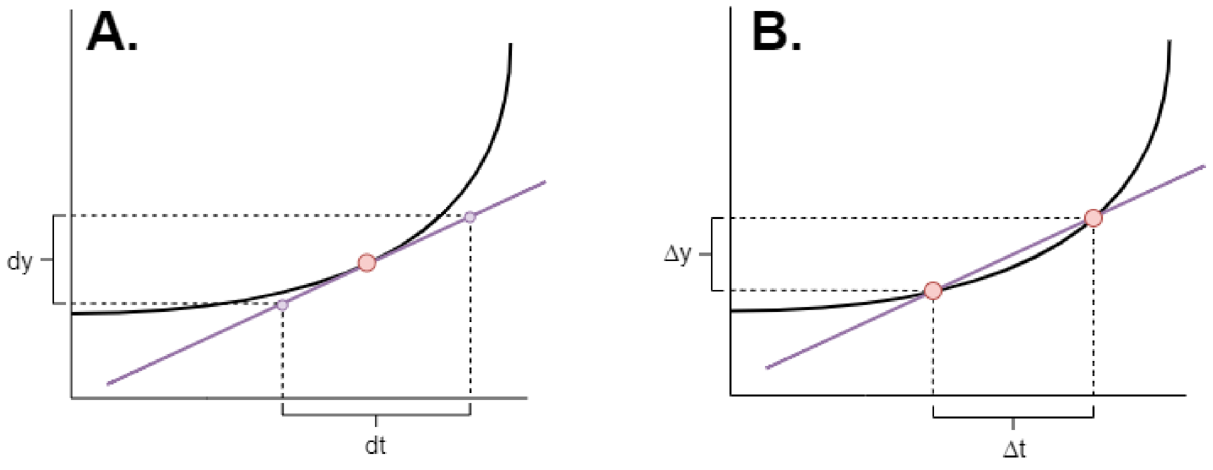

Figure 2. Instantaneous rate of change (A) as used in the joint model *versus* absolute rate of change (B).

The estimated joint model can be used to make individualized predictions. Based on a set of repeated measurements of the marker and relevant baseline covariates, the model can make predictions on future levels of the marker, and, more interestingly, on the probabilities of a future event. With graphs, it can be shown directly how adding new marker information updates the event probability of an individual. This is demonstrated in Figure 3 in the manuscript for two individuals. Each individual has several plots, with an increasing number of cardiac troponin measurements. On the left part of each plot, the concentrations are plotted with the estimated trajectory through them. The right part of each plot shows the corresponding predicted event probabilities for that individual, which are equal to one minus the survival probabilities. The shaded grey area represents the 95% confidence interval.

Joint model *versus* Time-Dependent Cox model

The joint model offers several advantages over traditional approaches for analyzing time-to-event data, such as Cox regression with a time-dependent covariate. For instance, the time-dependent cox model assumes that the level of a covariate stays constant in between two measurements when comparing marker levels of patients with and without event. In contrast, the joint model uses a separate regression model to describe the evolution of the marker over time and uses this estimated evolution in a time-to-event relative risk model for the event of interest. Additionally, the time-dependent cox model assumes that the availability of a measurement is not related to the event status. This indicates that the longitudinal marker needs to be an exogenous or external variable. However, cardiac troponin is a measurement taken from the individual and is, therefore, an endogenous or internal variable. The joint model is a more appropriate model compared to the time-dependent Cox model to analyze data with these features. Furthermore, as described above, an estimated joint model can be used to make individualized predictions on future levels of the marker and probabilities of a future event. The time-dependent cox model cannot be used to make survival predictions in a dynamic matter because the anticipated changes in future values of the time-varying covariate are not incorporated in calculating survival predictions for a single Cox model. In the primary analysis we have conducted joint modelling. For the external validation using HUNT, we were not able to conduct joint modelling, as we had a maximum number of two measurements per individual available. We attempted to fit our joint model to these data, but an accurate estimation of individual troponin trajectories is required to use this technique. Two measurements combined with a time gap of ten years between last measurement and end of follow-up precludes us to estimate the evolution of cardiac troponin accurately. We were therefore restricted to evaluate the relationship with serial cardiac troponin testing and cardiovascular death using Time-Dependent Cox regression analysis.

Table S1. Clinical characteristics of study population at first, second and third troponin measurement

Clinical characteristic	1st troponin measurement (n = 7,293)	2nd troponin measurement (n = 5,818)	3rd troponin measurement (n = 4,045)
Sex (female)	2,142 (29.4%)	1,619 (27.8%)	1,090 (26.9%)
Age (years)	58 (7)	66 (6)	70 (6)
Ethnic origin (other than white)	625 (8.6%)	448 (7.7%)	273 (6.8%)
Diabetes mellitus (yes)	333 (4.6%)	604 (10.4%)	357 (8.8%)
Systolic blood pressure (mmHg)	124 (17)	125 (16)	128 (16)
Total cholesterol (mmol/L)	5.8 (1.1)	5.2 (1.1)	5.1 (1.1)
High-density lipoprotein (mmol/L)	1.5 (0.4)	1.6 (0.5)	1.7 (0.5)
Low-density lipoprotein (mmol/L)	3.7 (1.0)	3.0 (1.0)	2.9 (1.0)
Smoker			
Never	3,451 (48.5%)	2,694 (47.7%)	1,874 (48.4%)
Ex-smoker	3,000 (42.2%)	2,683 (47.5%)	1,896 (48.9%)
Current	665 (9.3%)	268 (4.7%)	105 (2.7%)
Body mass index (kg/m^2)	26.4 (4.2)	26.7 (4.4)	26.5 (4.3)
Lipid-modifying medication (yes)	616 (8.5%)	2,008 (34.6%)	1,670 (41.3%)
Antihypertensive medication (yes)	1,267 (17.5%)	2,127 (36.6%)	1,664 (41.1%)
ACE inhibitors (yes)	561 (7.8%)	1,362 (23.4%)	1,128 (27.9%)
Antiplatelets (yes)	518 (7.2%)	1,390 (23.9%)	938 (23.2%)
Betablockers (yes)	448 (6.2%)	603 (10.4%)	433 (10.7%)

Continuous variables are presented as mean (SD). Categorical variables are presented as number (%).

Table S2. Association between clinical characteristics and longitudinal cardiac troponin I

Clinical characteristic	Estimate (95% CI)	P-value
Sex (female)	0.66 (0.63 to 0.68)	<0.001
Age (per 10 years)	1.32 (1.28 to 1.35)	<0.001
Ethnic original (White)	0.96 (0.93 to 1.06)	0.890
Diabetes Mellitus (yes)	1.22 (1.12 to 1.33)	<0.001
Systolic blood pressure (per 10 mmHg)	1.08 (1.07 to 1.10)	<0.001
Total cholesterol (mmol/L)	0.96 (0.94 to 0.97)	<0.001
High-density lipoprotein (mmol/L)	0.92 (0.88 to 0.96)	<0.001
Low-density lipoprotein (mmol/L)	0.96 (0.94 to 0.98)	<0.001
Current smoker (yes)	0.88 (0.83 to 0.93)	<0.001
Body mass index (per 5 kg/m^2)	1.07 (1.05 to 1.10)	<0.001

The estimate represents the univariable association of the risk factor with cardiac troponin.

Table S3. Baseline characteristics of those who experienced an event

Clinical characteristic	Cardiovascular death (n = 281)	Cardiac death (n = 143)	Non-cardiovascular death (n = 914)	All-cause death (n = 1,195)
Sex (female)	76 (27.0%)	26 (18.2%)	270 (29.5%)	346 (29.0%)
Age (years)	61 (6)	60 (6)	61 (7)	61 (7)
Ethnic origin (other than white)	40 (14.2%)	20 (14.0%)	80 (8.8%)	120 (10.0%)
Diabetes mellitus (yes)	31 (11.0%)	14 (9.8%)	62 (6.8%)	93 (7.8%)
Systolic blood pressure (mmHg)	129 (18)	131 (17)	126 (18)	127 (18)
Total cholesterol (mmol/L)	5.9 (1.2)	5.9 (1.1)	5.8 (1.1)	5.9 (1.1)
High-density lipoprotein (mmol/L)	1.4 (0.4)	1.4 (0.4)	1.5 (0.4)	1.5 (0.4)
Low-density lipoprotein (mmol/L)	3.8 (1.0)	3.8 (1.0)	3.7 (1.0)	3.8 (1.0)
Smoker				
Never	123 (44.9%)	56 (39.7%)	364 (40.6%)	487 (41.6%)
Ex-smoker	115 (42.0%)	66 (46.8%)	401 (44.7%)	516 (44.1%)
Current	36 (13.1%)	19 (13.5%)	132 (14.7%)	168 (14.3%)
Body mass index (kg/m^2)	27.5 (4.9)	27.6 (4.3)	26.7 (4.6)	26.9 (4.7)
Lipid-modifying medication (yes)	31 (11.2%)	21 (14.7%)	75 (8.3%)	106 (9.0%)
Antihypertensive medication (yes)	102 (36.8%)	57 (39.9%)	192 (21.2%)	294 (24.9%)
ACE inhibitors (yes)	36 (13.0%)	20 (14.0%)	77 (8.5%)	113 (9.6%)
Antiplatelets (yes)	45 (16.2%)	31 (21.7%)	80 (8.8%)	125 (10.6%)
Betablockers (yes)	39 (14.1%)	20 (14.0%)	72 (7.9%)	111 (9.4%)
Baseline cardiac troponin I level (ng/L)	4.8 [3.0 to 9.3]	5.4 [3.1 to 11.3]	3.4 [2.3 to 5.4]	3.6 [2.4 to 6.0]

Continuous variables are presented as mean (SD) or median (IQR), as appropriate. Categorical variables are presented as number (%). Abbreviations: ACE, Angiotensin-converting enzyme.

Table S4. Association between the cardiac troponin I level at baseline and cardiovascular death

	HR (95% CI)	P-value
**Crude Model**		
Cardiac troponin I, per doubling increase	1.44 (1.36 to 1.53)	<0.001
**Adjusted model (age and sex)**		
Cardiac troponin I, per doubling increase	1.37 (1.28 to 1.46)	<0.001
Age, years	1.11 (1.10 to 1.14)	<0.001
Sex, female	1.02 (0.78 to 1.33)	0.897
**Adjusted model (known CVD risk factors[†])**		
Cardiac troponin I, per doubling increase	1.34 (1.25 to 1.43)	<0.001
Age, years	1.11 (1.09 to 1.13)	<0.001
Sex, female	1.00 (0.76 to 1.31)	0.998
Diabetes mellitus, yes	2.71 (1.82 to 4.06)	<0.001
Total cholesterol, mmol/L	1.03 (0.69 to 1.52)	0.894
High-density lipoprotein, mmol/L	0.75 (0.51 to 1.12)	0.158
Low-density lipoprotein, mmol/L	1.06 (0.68 to 1.64)	0.795
Systolic blood pressure, 10 mmHg	1.10 (1.02 to 1.18)	0.012
Smoking status, current	2.00 (1.42 to 2.83)	<0.001

[†]The model adjusted for known cardiovascular risk factors included age, sex, diabetes mellitus, total cholesterol, high-density lipoprotein, low-density lipoprotein, systolic blood pressure, smoking status and baseline cardiac troponin measurements.

Table S5. Baseline characteristics of individuals without cardiac disease at baseline

Clinical characteristic	Study population (n = 6,773)	No cardiovascular death (n = 6,549)	Cardiovascular death (n = 224)
Sex (female)	2,002 (29.6%)	1,936 (29.6%)	66 (29.5%)
Age (years)	57 (7)	57 (7)	61 (6)
Ethnic origin (other than white)	546 (8.1%)	514 (7.9%)	32 (14.3%)
Diabetes mellitus (yes)	275 (4.1%)	252 (3.8%)	23 (10.3%)
Systolic blood pressure (mmHg)	124 (17)	123 (17)	131 (17)
Total cholesterol (mmol/L)	5.8 (1.1)	5.8 (1.1)	6.0 (1.2)
High-density lipoprotein (mmol/L)	1.5 (0.4)	1.5 (0.4)	1.4 (0.4)
Low-density lipoprotein (mmol/L)	3.7 (1.0)	3.7 (1.0)	3.9 (1.0)
Smoker			
Never	3,234 (48.9%)	3,135 (49.0%)	99 (45.2%)
Ex-smoker	2,755 (41.7%)	2,667 (41.7%)	88 (40.2%)
Current	624 (9.4%)	592 (9.3%)	32 (14.6%)
Body mass index (kg/m^2)	26.3 (4.1)	26.2 (4.1)	27.4 (4.6)
Lipid-modifying medication (yes)	430 (6.4%)	420 (6.5%)	10 (4.5%)
Antihypertensive medication (yes)	989 (14.7%)	925 (14.2%)	64 (29.1%)
ACE inhibitors (yes)	428 (6.4%)	407 (6.3%)	21 (9.5%)
Antiplatelets (yes)	278 (4.1%)	265 (4.1%)	13 (5.9%)
Betablockers (yes)	334 (5.0%)	307 (4.7%)	27 (12.3%)
Baseline cardiac troponin I level (ng/L)	3.2 [2.2 to 5.0]	3.2 [2.2 to 5.0]	3.9 [2.8 to 7.0]

Continuous variables are presented as mean (SD) or median (IQR), as appropriate. Categorical variables are presented as number (%). Abbreviations: ACE, Angiotensin-converting enzyme.

Table S6. Association between the temporal evolution of cardiac troponin I and cardiovascular death in individuals without cardiac disease at baseline

	**HR (95% CI)**	**P-value**
**Crude Model**		
Temporal evolution of cardiac troponin I*		
Level	1.43 (1.19 to 1.68)	<0.001
Slope	1.74 (0.58 to 4.94)	0.336
Area	1.43 (1.19 to 1.68)	<0.001
**Adjusted model (age and sex)**		
Temporal evolution of cardiac troponin I*		
Level	1.37 (1.24 to 1.52)	<0.001
Slope	1.88 (0.64 to 5.32)	0.254
Area	1.36 (1.21 to 1.51)	<0.001
Age, years	1.11 (1.08 to 1.14)	<0.001
Sex, female	1.10 (0.79 to 1.49)	0.525
**Adjusted model (known CVD risk factors †)**		
Temporal evolution of cardiac troponin I*		
Level	1.34 (1.20 to 1.49)	<0.001
Slope	1.94 (0.67 to 5.70)	0.210
Area	1.32 (1.18 to 1.48)	<0.001
Age, years	1.10 (1.07 to 1.14)	<0.001
Sex, female	1.07 (0.77 to 1.48)	0.699
Diabetes mellitus, yes	2.50 (1.56 to 3.82)	<0.001
Total cholesterol, mmol/L	1.09 (0.96 to 1.22)	0.163
High-density lipoprotein, mmol/L	0.77 (0.54 to 1.09)	0.143
Systolic blood pressure, 10 mmHg	1.16 (1.07 to 1.24)	<0.001
Smoking status, current	2.03 (1.37 to 2.99)	<0.001

* Hazard ratios (HRs) and 95% confidence intervals (Cis) are given per doubling in cardiac troponin in level, slope (delta of the cardiac troponin's levels/ 5 year) and area under the trajectory of cardiac troponin. †The model adjusted for known cardiovascular risk factors included age, sex, diabetes mellitus, total cholesterol, high-density lipoprotein, systolic blood pressure, smoking status and serial cardiac troponin measurements. Abbreviations: CVD, cardiovascular disease.

Table S7. The longitudinal cardiac troponin's accuracy

	Risk Time Window	AUC (t)
**Crude Model**	2 years	0.644
	5 years	0.673
**Adjusted model (age and sex)**	2 years	0.774
	5 years	0.786
**Adjusted model (known CVD risk factors)** *	2 years	0.794
	5 years	0.792

We determined the longitudinal cardiac troponin's predictive accuracy (i.e., an ability of cardiac troponin to discriminate between an individual who died due to cardiovascular disease within a given risk time window after the last measurement, and the individual who does not experience the event within the same risk time window) using the time-dependent area under the curve. For this purpose, we chose the first 16 years as the collection time period, and we assessed risk time windows at 2 and 5 years after collection time. We determined the predictive accuracy of the cardiac troponin's levels in univariable and multivariable adjusted non-competing risk models. *The model adjusted for known cardiovascular risk factors included age, sex, diabetes mellitus, total cholesterol, high-density lipoprotein, systolic blood pressure, smoking status and serial cardiac troponin measurements. Abbreviations: AUC, area under the curve; CVD, cardiovascular disease.

Table S8. Association between serial cardiac troponin measurements and cardiovascular death in HUNT

	HR (95% CI)	P-value
**Crude Model**		
Cardiac troponin I, per 1-SD doubling increase	1.84 (1.79 to 1.90)	<0.001
**Adjusted model (age and sex)**		
Cardiac troponin I, per 1-SD doubling increase	1.35 (1.27 to 1.42)	<0.001
Age, years	1.14 (1.13 to 1.15)	<0.001
Sex, female	1.49 (1.30 to 1.71)	<0.001
**Adjusted model (known CVD risk factors[†])**		
Cardiac troponin I, per 1-SD doubling increase	1.33 (1.25 to 1.40)	<0.001
Age, years	1.14 (1.13 to 1.15)	<0.001
Sex, female	1.40 (1.21 to 1.61)	<0.001
Diabetes mellitus, yes	1.57 (1.25 to 1.98)	<0.001
Total cholesterol, mmol/L	0.78 (0.54 to 1.11)	0.167
High-density lipoprotein, mmol/L	1.01 (0.73 to 1.40)	0.954
Low-density lipoprotein, mmol/L	1.24 (0.85 to 1.82)	0.258
Systolic blood pressure, 10 mmHg	1.05 (1.02 to 1.08)	<0.001
Smoking status, current	1.57 (1.34 to 1.85)	<0.001

The table shows the results of the time-dependent Cox regression analysis evaluating serial troponin measurements in relation to cardiovascular death in the external validation cohort. [†]The model adjusted for known cardiovascular risk factors included age, sex, diabetes mellitus, total cholesterol, high-density lipoprotein, low-density lipoprotein, systolic blood pressure, smoking status and baseline cardiac troponin measurements.

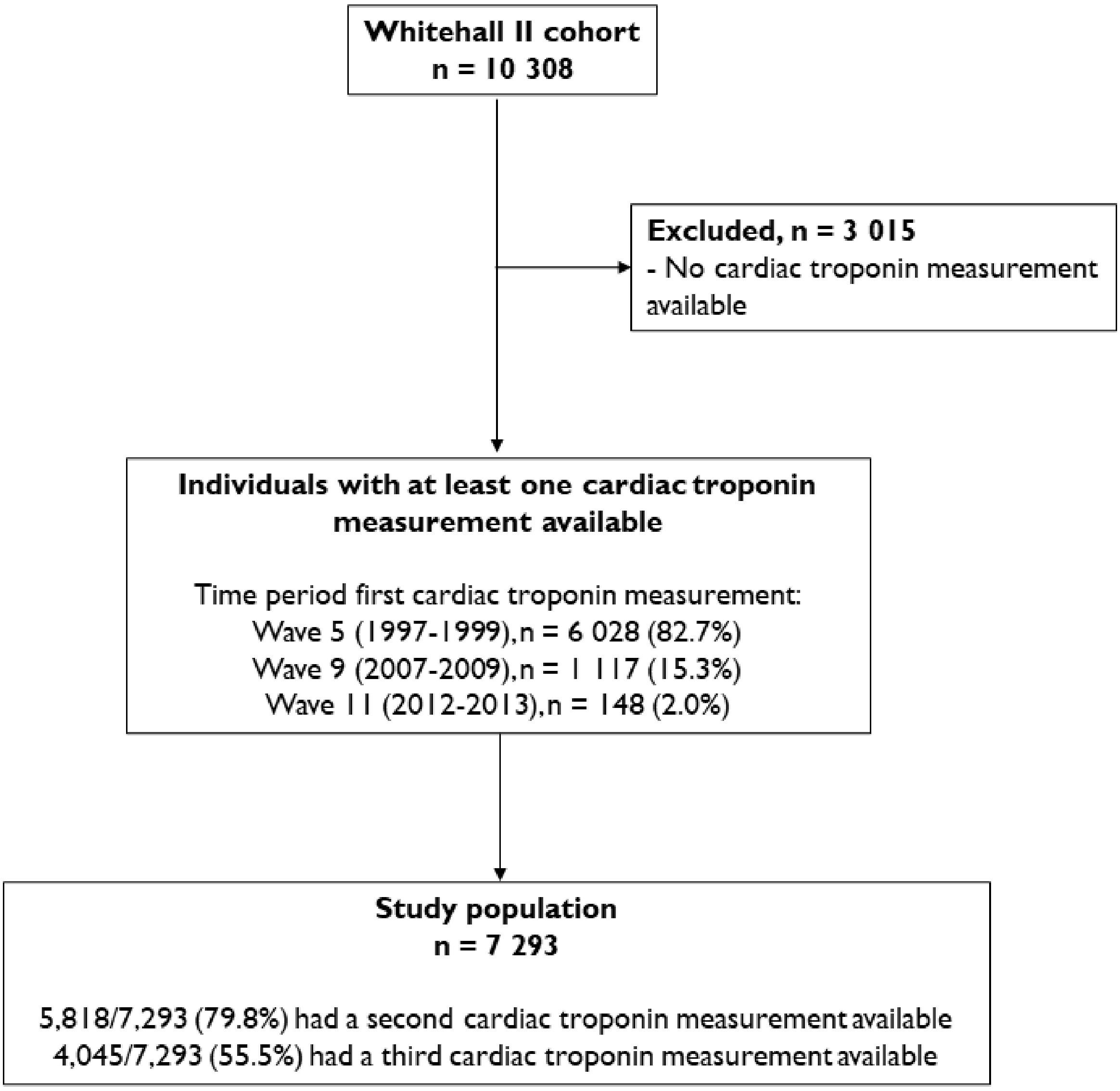

Fig. S1 Study flow diagram.

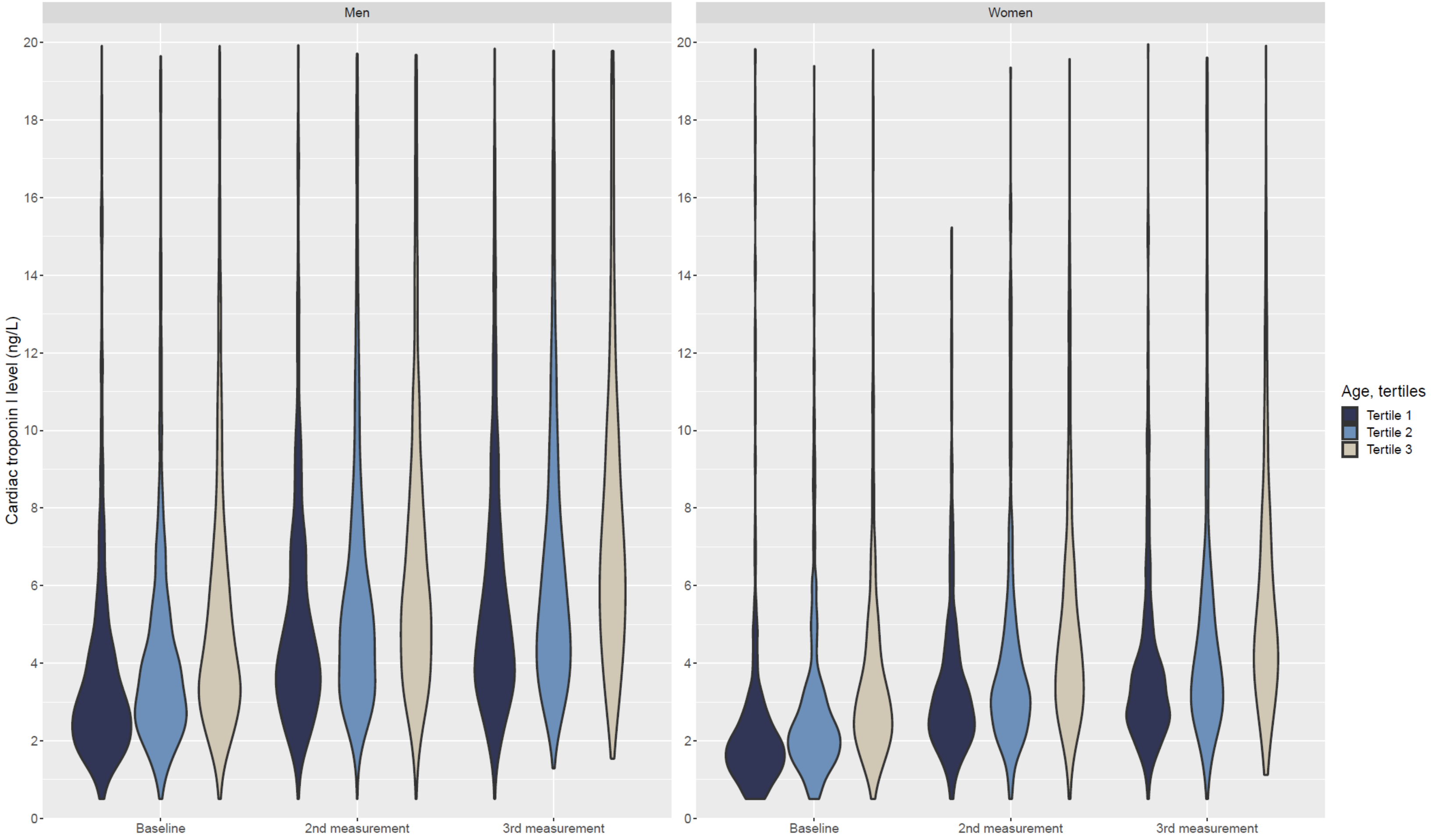

Fig. S2 Cardiac troponin I levels at baseline, 10 years and 15 years, stratified by sex and age groups. Age groups are defined by tertiles (tertile 1: 44.8 to 52.2 years of age ; tertile 2: 52.3 to 60.1 years of age; tertile 3: 60.2 to 82.1 years of age). Individuals with a second troponin measurement, n=5,818, median (25th to 75th percentile) time between baseline and second troponin measurement: 10.4 (10.0-10.8) years. Individuals with a third troponin measurement, n=4,045, median (25th to 75th percentile) time between baseline and third troponin measurement: 14.6 (14.4-14.6) years.

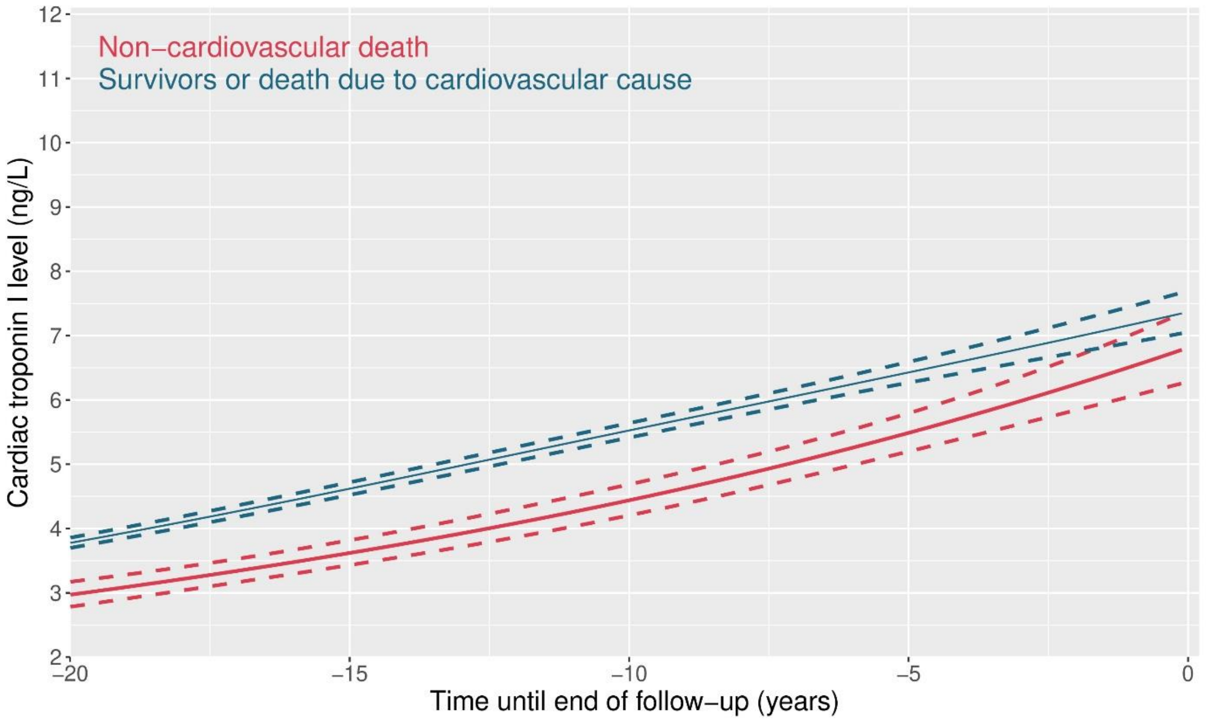

Fig. S3 Trajectories of cardiac troponin I with 95% confidence intervals before non-cardiovascular death occurred or at end of follow-up. The red line refers to the average troponin trajectory of those individuals who died due to non-cardiovascular causes, and the blue line refers to the average troponin trajectory of those individuals who survived or died due to cardiovascular causes. Estimates are adjusted for sex and age.

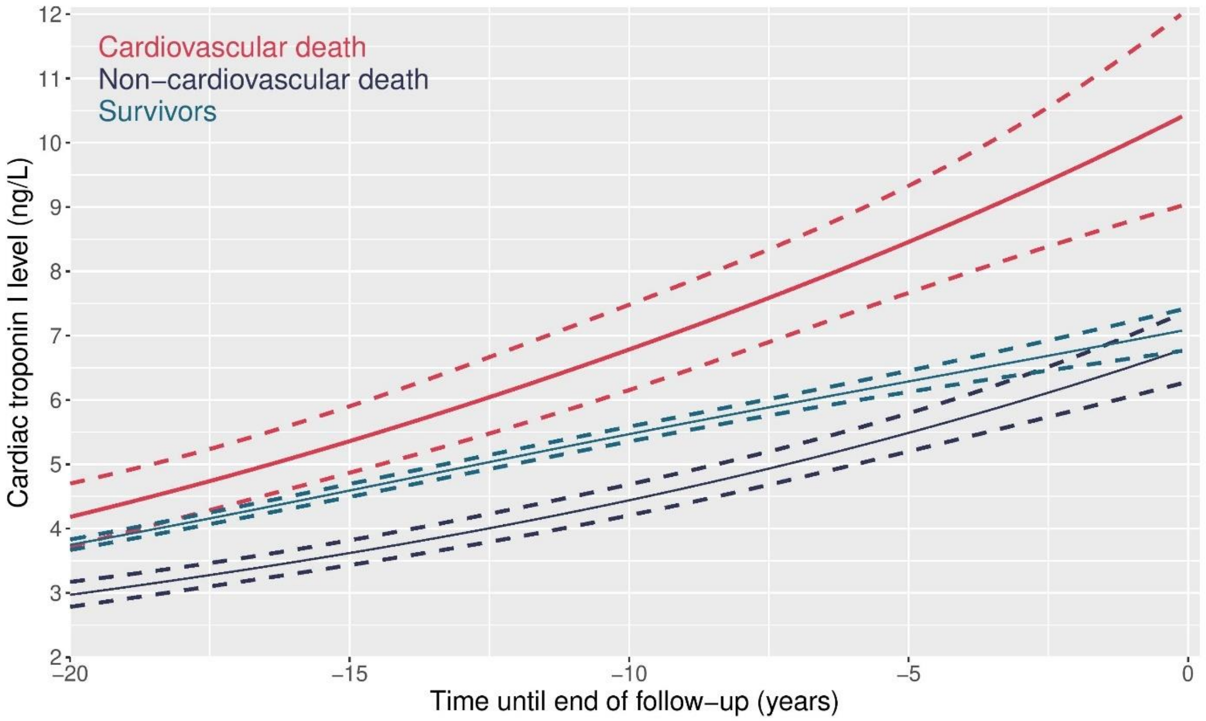

Fig. S4 Trajectories of cardiac troponin I with 95% confidence intervals before cardiovascular death or non-cardiovascular death occurred or at end of follow-up. The red line refers to the average troponin trajectory of those individuals who died due to cardiovascular causes, the light blue line refers to the average troponin trajectory of those individuals who survived, and the dark blue line refers to the average troponin trajectory of those individuals who died due to non-cardiovascular causes. Estimates are adjusted for sex and age.

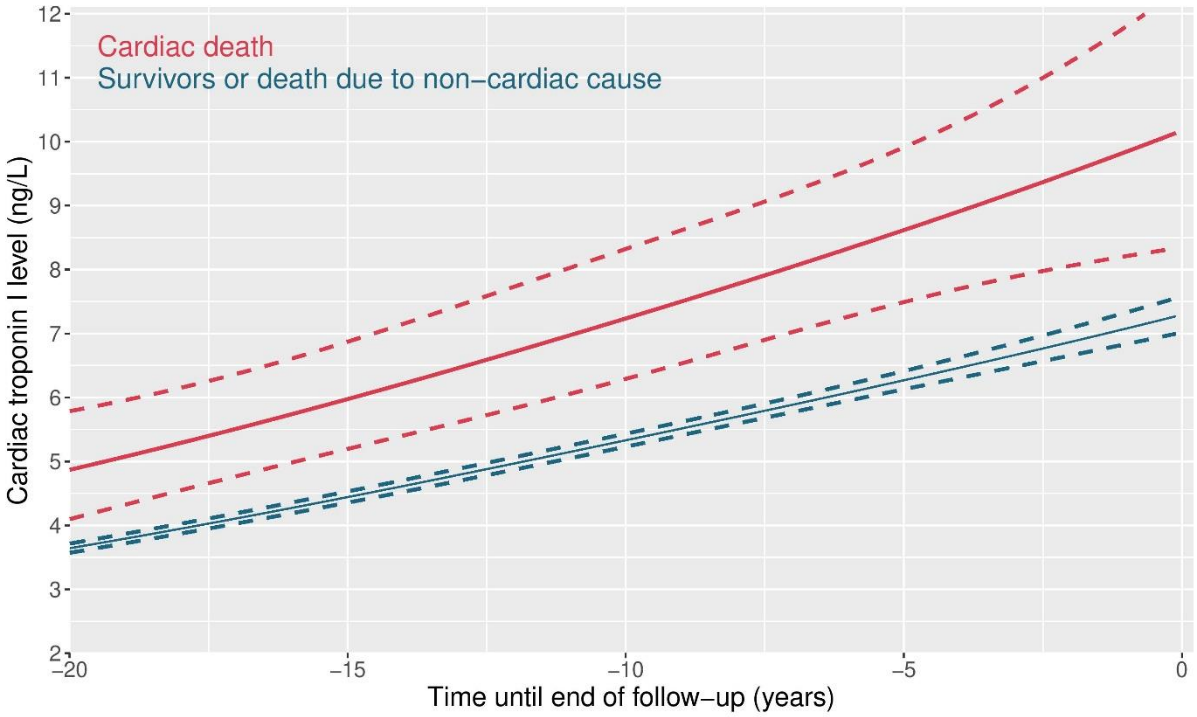

Fig. S5 Trajectories of cardiac troponin I with 95% confidence intervals before cardiac death occurred or at end of follow-up. The red line refers to the average troponin trajectory of those individuals who died due to cardiac causes, and the blue line refers to the average troponin trajectory of those individuals who survived or died due to non-cardiac causes. Estimates are adjusted for sex and age.

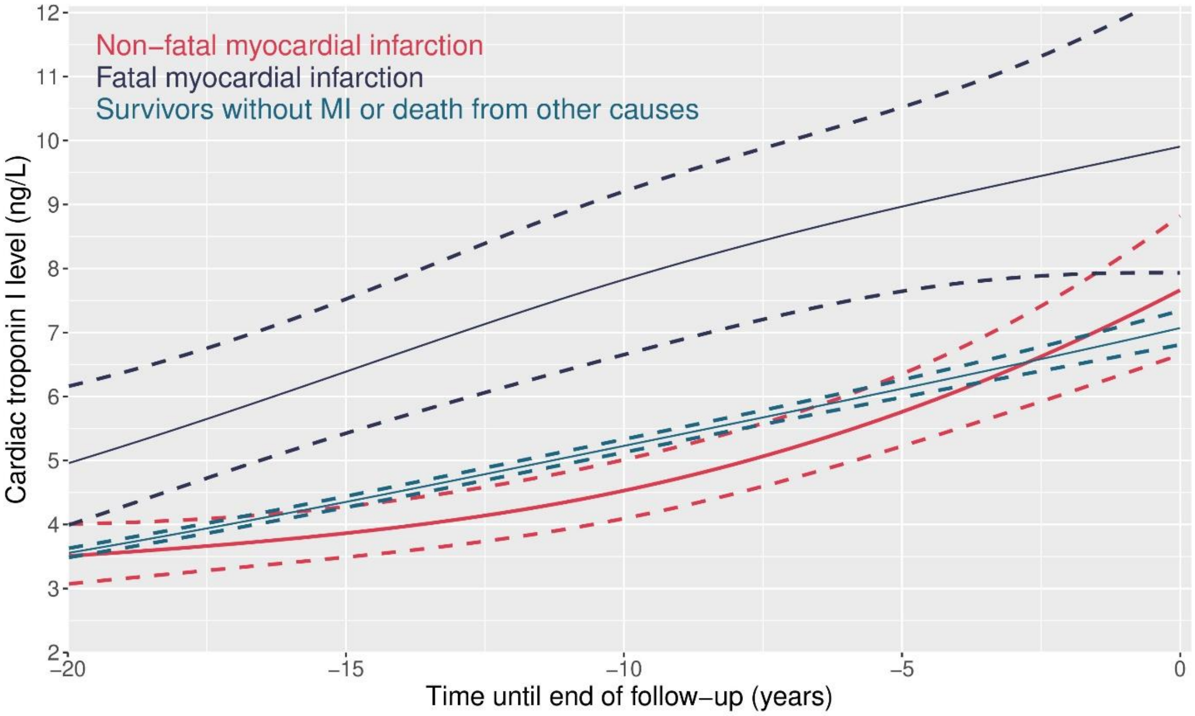

Fig. S6 Trajectories of cardiac troponin I with 95% confidence intervals before non-fatal myocardial infarction, fatal myocardial infarction and no myocardial infarction event. The red line refers to the average troponin trajectory of those individuals who experienced a non-fatal myocardial infarction, the dark blue line refers to the average troponin trajectory of those individuals who experienced a fatal myocardial infarction, and the light blue line refers to the average troponin trajectory of those individuals who survived without myocardial infarction or died from other causes. Estimates are adjusted for sex and age. MI=myocardial infarction.

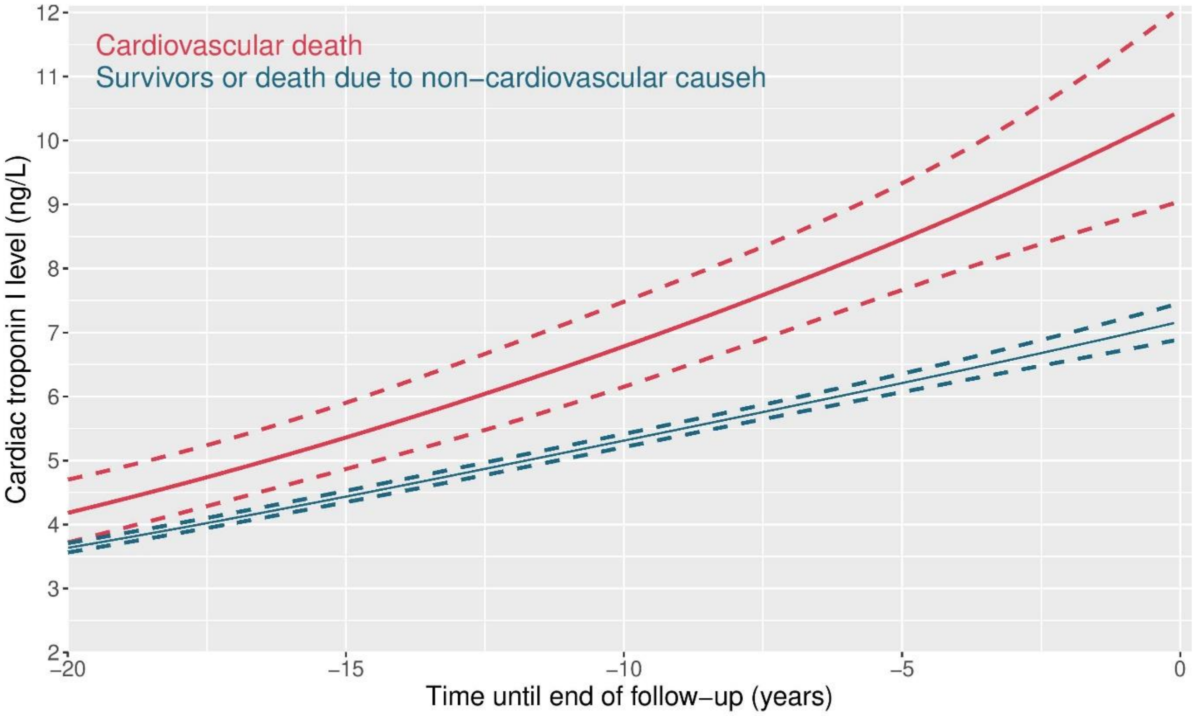

Fig. S7 Trajectories of cardiac troponin I with 95% confidence intervals in individuals without baseline cardiac disease before cardiovascular death occurred or at end of follow-up. The red line refers to the average troponin trajectory of those individuals who died due to cardiovascular causes, and the blue line refers to the average troponin trajectory of those individuals who survived or died due to non-cardiovascular causes. Estimates are adjusted for sex and age.

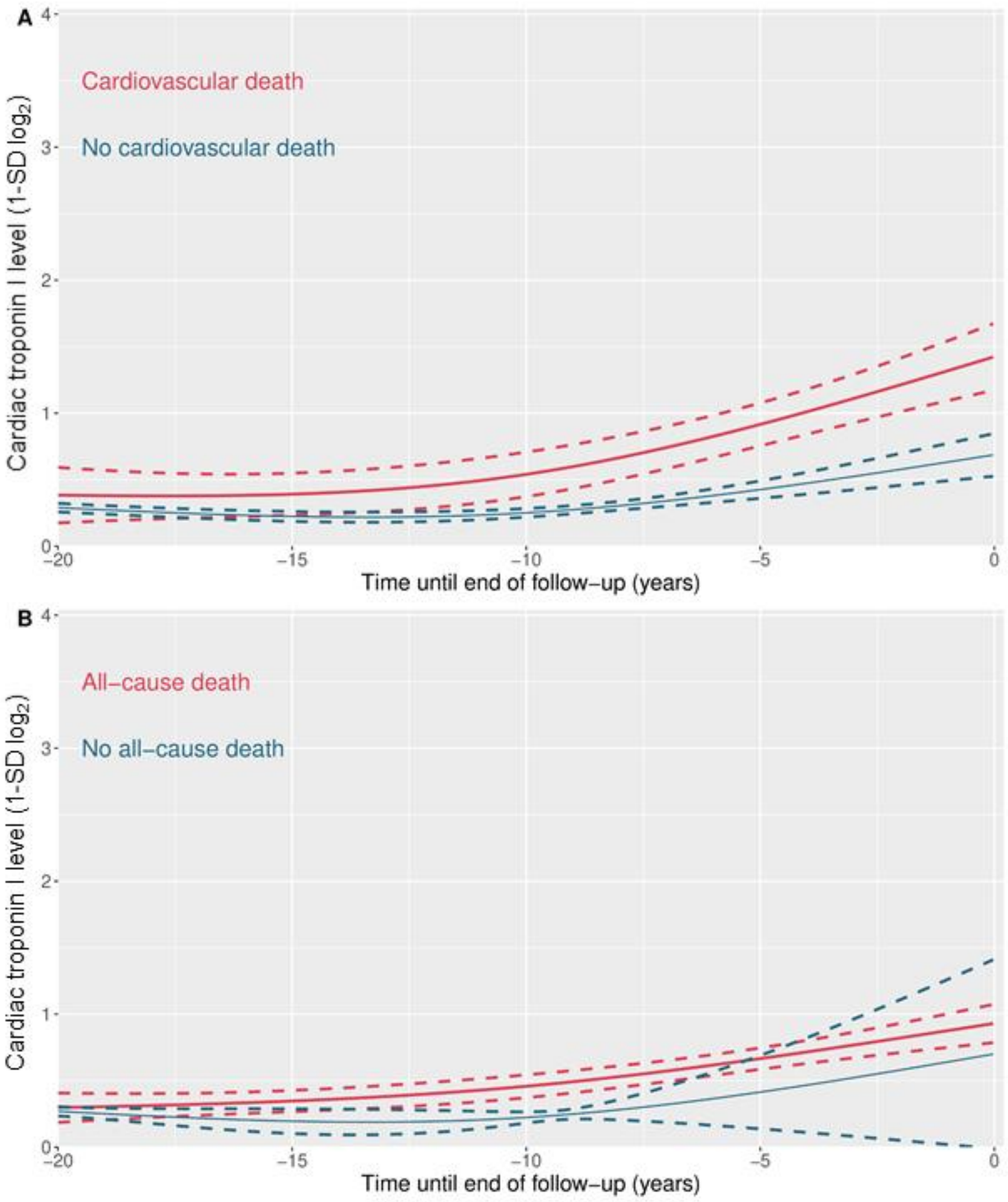

Fig. S8 Trajectories of cardiac troponin I with 95% confidence intervals before cardiovascular death (Panel A) and death from any cause (Panel B) in HUNT. In Panel A, the red line refers to the average troponin trajectory of those individuals who died due to cardiovascular causes, and the blue line refers to the average troponin trajectory of those individuals who survived or died due to non-cardiovascular causes. In Panel B, the red line refers to the average troponin trajectory of those individuals who died from any cause, and the blue line refers to the average troponin trajectory of those individuals who survived. Estimates are adjusted for sex and age.
